# No Evidence for the Involvement of Leiomodin-1 Antibodies in the Pathogenesis of Onchocerciasis-Associated Epilepsy

**DOI:** 10.3390/pathogens10070845

**Published:** 2021-07-05

**Authors:** An Hotterbeekx, Melissa Krizia Vieri, Melanie Ramberger, Ashraf Jozefzoon-Aghai, Michel Mandro, Floribert Tepage, Alfred Dusabimana, Samir Kumar-Singh, Maarten J. Titulaer, Robert Colebunders

**Affiliations:** 1 Global Health Institute, University of Antwerp, 2610 Antwerp, Belgium; krizia.vieri@uantwerpen.be (M.K.V.); alfred.dusabimana@uantwerpen.be (A.D.); robert.colebunders@uantwerpen.be (R.C.); 2 Molecular Pathology Group, Laboratory of Cell Biology and Histology, University of Antwerp, 2610 Antwerp, Belgium; Samir.kumarsingh@uantwerpen.be; 3 Department of Neurology, Erasmus MC University Medical Center, 3015 Rotterdam, The Netherlands; ramberger.melanie@gmail.com (M.R.); a.aghai@erasmusmc.nl (A.J.-A.); m.titulaer@erasmusmc.nl (M.J.T.); 4 Provincial Health Division of Ituri, Ministry of Health, Bunia, Democratic Republic of the Congo; michelmandro8@gmail.com; 5 Ministry of Health, Buta, Democratic Republic of the Congo; floritepage@yahoo.fr

**Keywords:** onchocerciasis-associated epilepsy, nodding syndrome, leiomodin-1, autoimmune

## Abstract

Nodding syndrome has been suggested to be triggered by neurotoxic leiomodin-1 auto-antibodies cross-reacting with *Onchocerca volvulus*. Here, we screened serum and CSF samples of persons with nodding syndrome and other forms of onchocerciasis-associated epilepsy (OAE) and African and European controls for leiomodin-1 antibodies by a cell-based assay (CBA) and Western blot (WB). These samples were also investigated for the presence of auto-antibodies cross-reacting with rat brain tissue by immunohistochemistry (IHC). Additionally, IHC was used to detect the leiomodin-1 protein in post-mortem brain samples of persons with OAE who died. Leiomodin-1 antibodies were detected by CBA in 6/52 (12%) and by WB in 23/54 (43%) persons with OAE compared to in 14/61 (23%) (*p =* 0.113) and 23/54 (43%) *(p =* 0.479) of controls without epilepsy. Multivariable exact logistic regression did not show an association between *O. volvulus* infection or epilepsy status and the presence of leiomodin-1. Leiomodin-1 antibodies were not detected in 12 CSF samples from persons with OAE or in 16 CSF samples from persons with acute-onset neurological conditions, as well as not being detected in serum from European controls. Moreover, the leiomodin-1 protein was only detected in capillary walls in post-mortem brain tissues and not in brain cells. IHC on rat brain slides with serum samples from persons with OAE or controls from persons with or without *O. volvulus* infection revealed no specific staining pattern. In conclusion, our data do not support OAE to be an autoimmune disorder caused by leiomodin-1 antibodies.

## 1. Introduction

Nodding syndrome and other forms of onchocerciasis-associated epilepsy (OAE) are characterized by an onset of seizures between the age of 3 and 18 years [1]. The pathological mechanism of this type of epilepsy remains unknown but seems to be dependent on the *O. volvulus* microfilarial load. This suggests that *O. volvulus* may directly or indirectly trigger epilepsy. However, no parasites have been detected in the cerebrospinal fluid (CSF) or brains of people with OAE, indicating that parasites are not likely to invade the central nervous system directly [2,3]. Moreover, post-mortem brains of people who died with nodding syndrome and other forms of OAE showed signs of neuro-inflammation and deposition of tau-reactive neurofibrillary tangles and threads, but no evidence of a parasitic infection [2,4]. It has been suggested that OAE is an autoimmune disease caused by cross-reacting antibodies against *O. volvulus* tropomyosin and human leimodin-1 [5,6]. This hypothesis is based on antibodies reacting with leiomodin-1 which have been detected more frequently in the serum and CSF of persons with nodding syndrome compared to healthy village controls, and cross-reacting with *O. volvulus* lysates [5].

Leiomodin-1 is an intracellular protein containing an actin-binding domain and is expressed in various smooth muscle cells, including endothelial cells, the intestinal wall and the bladder [7,8]. Leiomodin-1 shows similarity to the tropomodulins, but, currently, very limited information is available on the function of leiomodin-1, and the transcription profile is incomplete [7]. Recently, the presence of leiomodin-1 has been shown in the hippocampus and cerebellum of the brain of mouse brains [5], whereas the production in the human brain is limited [9]. Investigation of the Human Protein Atlas shows that leiomodin-1 is mainly expressed in endothelial cells in the human brain and in a limited amount of positive neurons [9,10]. However, a recent study suggested that leiomodin-1 was expressed on the membrane of newly formed neurons, but not on neural progenitor cells or mature neurons [6]. Moreover, leiomodin-1 antibodies were only toxic to cells expressing leiomodin-1 on the membrane [6].

There are also several arguments against the hypothesis that nodding syndrome is an autoimmune disease caused by leiomodin-1 antibodies. First, these antibodies have only been detected in half of the children with nodding syndrome (53%) and were also present in 31% of controls [5,11]. Furthermore, leiomodin-1 was shown to be most abundant in the hippocampus region of the mouse brain [5], whereas post-mortem brains showed no overt pathological changes in the hippocampus [2,4]. Moreover, these leiomodin-1 antibodies could be markers of disease propagation and not markers of disease initiation. In conclusion, the role of leiomodin-1 antibodies in the development of nodding syndrome remains unclear.

Therefore, this study aimed to identify leiomodin-1 antibodies in the serum and CSF of persons with nodding syndrome and other forms of OAE from the DRC and South Sudan. We also screened these samples for the presence of other antibodies cross-reacting with the central nervous system. In addition, we studied leiomodin-1 expression in various in vitro neuronal cells and post-mortem brains of persons with OAE who died.

## 2. Materials and Methods

### 2.1. Study Participants and Sample Collection

Serum samples were obtained from two village matched case–control studies in the DRC to identify risk factors for epilepsy in onchocerciasis-endemic areas, one in Ituri [12] and one in Bas Uéle [13], a clinical trial assessing the effect of ivermectin on the frequency of seizures in *O. volvulus*-infected persons with epilepsy [14], controls with and without *O. volvulus* infection from a rapid epidemiological mapping of onchocerciasis (REMO) study in Ituri [15] and a diagnostic study among patients admitted to the Mosango general hospital, in Kwilu Province [16] (Table 1). CSF samples of cases were obtained from persons with OAE from Maridi, an onchocerciasis-endemic region in South Sudan, and from persons without *O. volvulus* infection with acute-onset neurological conditions from the Mosango general hospital in Kwilu, DRC (Table 2).

A person was considered to present OAE if he/she had a previously healthy child, lived in an onchocerciasis meso- or hyper-endemic area and developed epilepsy between the ages of 3 and 18 years without any obvious reason (criteria of the OAE definition) [17]. Onchocerciasis antibodies were detected using the OV16 rapid diagnostic test (OV16 RDT, SD Bioline Onchocerciasis IgG4 rapid test, Abbott Standard Diagnostics, Inc., Yongin, Republic of Korea). For comparison, 16 Dutch sera were tested as controls (12 patients with chronic focal epilepsy without antibodies associated with encephalitis, and 4 healthy persons) [18]. All 12 persons with OAE from Maridi had high mf densities (>80 mf/skin snip), but in none of the samples was DNA of *O. volvulus*, nor *Wolbachia,* the bacterial endosymbiont, found [3].

### 2.2. Detection of Anti-Leiomodin-1 Antibodies in a Cell-Based Assay

Screening for the presence of anti-leiomodin-1 antibodies was conducted in serum (dilution 1:40) and CSF (dilution 1:2) of cases and controls using HEK-293 cells expressing recombinant leiomodin-1. HEK-293 cells were cultured in Dulbecco’s modified Eagle medium (Dulbecco) for 1 day and subsequently transfected with a plasmid containing GFP-tagged recombinant leiomodin-1 (origene RG209941, vector pCMV-AC with ampicillin resistance marker) for 3 min in FuGENE HD transfection reagent (Promega). Transfected cells were fixed with 4% paraformaldehyde and permeabilized with 0.2% Triton in PBS. Fixed cells were first incubated with 0.5% BSA, and consecutively with samples or primary anti-leiomodin-1 antibody as a positive control (ab104858, Protech 15117-1) for 1 h at room temperature, followed by staining with cy3-conjugated donkey anti-human antibody (Jackson Immuno Research 709-166-149) for 1 h at room temperature. Serum from a healthy European volunteer was used as a background control. Slides were mounted with Vectashield (H-1500 hard set), and cells were visualized by fluorescence microscopy (Nikon eclipse 80i).

### 2.3. Detection of Anti-Leiomodin-1 Antibodies by Western Blot

In addition to the cell-based assay, serum and CSF were also screened for the presence of leiomodin-1 antibodies by Western blot. Recombinant leiomodin-1 was produced by transfected HEK293 cells (origene RG209941, vector pCMV-AC with ampicillin resistance marker). To extract the leiomodin-1 protein fraction, cells were first washed with phosphate buffered saline followed by lysis in lysis buffer containing 50 mM Tris HCl, 150 mM NaCl and 1 mM EDTA. Next, cells were disrupted by sonication and centrifuged. The supernatant containing the protein fraction from the cytoplasma was collected and stored separately. The remaining pellet containing the membrane protein was subjected to another lysis step with lysis buffer containing 50 mM Tris HCl, 150 mM NaCl, 1 mM EDTA and 1% triton-x-100:protease inhibitor (1:25). Proteins in each fraction were precipitated and quantified using the Pierce™ BCA Protein Assay Kit (Thermo Scientific, Waltham, MA, USA) according to the manufacturer’s instructions. Next, 2 µg of protein fraction was run on a NuPAGE polyacrylamide gel for 90 min and 100 V. Protein fractions were blotted in a nitrocellulose membrane for 60 min at 100 V. Nitrocellulose membranes were blocked with 5% bovine serum albumin, followed by incubation with the sample at 4 °C. Serum samples were diluted 1:500 prior to blotting, and CSF samples were diluted 1:50. Commercial primary anti-leiolodin-1 antibody (Rabbit polyclonal, abcam, ab104858) was diluted 1:500 and used as a positive control. Swine anti-rabbit HRP or polyclonal rabbit anti-human HRP (Dako) was used as secondary antibody at a dilution of 1:1000. The blots were developed using BM Chemiluminescence Western blotting substrate (Sigma Aldrich, Saint-Louis, MO, USA).

### 2.4. Immunohistochemistry to Screen for Auto-Antibodies in Serum Samples

To screen for the presence of auto-antibodies cross-reacting with brain tissue, rat brain slides were prepared for immunohistochemistry, as described before [19]. Briefly, 8 µm slides including an intact cerebellum and hippocampus were prepared in Superfrost Plus slides (Thermo Fisher Scientific, Waltham, MA, USA) and blocked with 1% bovine serum albumin in phosphate buffered saline for 30 min. Serum (diluted 1:200) or CSF (diluted 1:2) from cases and controls was applied on the brain slides and incubated overnight at 4 °C. After washing, slides were incubated with goat anti-human IgG (Heavy + Light, biotinylated; Vector Laboratories Inc., BA-3000; dilution 1:2000) for 2 h, followed by incubation with Avidin & Biotinylated horseradish peroxidase macromolecular Complex vectastain (Vector Laboratories Inc., Elite kit, Pk 6100, Burlingame, CA, USA). Slides were developed with 3,3′-Diaminobenzidine (Kit 6-SK-4105, Vector Laboratories Inc. (Brunschwig Chemie, Basel, Switzerland), counterstained with hematoxylin and examined by light microscopy (Olympus BX50F) for specific staining patterns, blinded for patient status.

### 2.5. Immunohistochemistry to Detect the Presence of Leiomodin-1 in Post-Mortem Brains of People with OAE Who Died

Immunohistochemistry was used to detect the presence of leiomodin-1 in post-mortem brains of people with OAE who died in northern Uganda (Table 3). The post-mortem material and the study population have been described before [2]. Formalin-fixed paraffin-embedded brain tissue slides were deparaffinized followed by antigen retrieval by boiling in citric acid buffer in a microwave. Endogenous peroxidases were blocked in H_2_O_2_, and non-specific binding sites were blocked in normal horse serum. Samples were incubated overnight with rabbit anti-leiomodin-1 antibody (1:100 dilution; ab 104858, abcam) at 4 °C. After washing, the slides were incubated with biotinylated Donkey anti-rabbit secondary antibody (dilution 1:200; Abcam) for 30 min and conjugated with Extravidin-peroxidase (Sigma Aldrich, Saint-Louis, MO, USA). The slides were developed with 3,3′-Diaminobenzidine, counterstained with hematoxylin and visualized by light microscopy. A formalin-fixed paraffin-embedded human colon was used as the positive control, and incubation with the secondary antibody was omitted as the negative control.

### 2.6. Statistics

Clinical and demographic variables were presented using the median and interquartile range (IQR) for continuous variables and absolute and relative frequencies for categorical variables. Fisher’s exact test was used to compare the categorical variables and Wilcoxon sum rank test for continuous variables. Multivariable exact logistic regression including age, gender, *O. volvulus* infection and epilepsy status was used to determine factors associated with the presence of leimodin-1. Data were analyzed using SAS version 9.4, SAS Institute Inc. Cary, NC, and R version 4.0.2. A two-sided 5% significance level was used.

## 3. Results

### 3.1. Leiomodin-1 Antibodies in Serum and CSF

**Figure 1:**
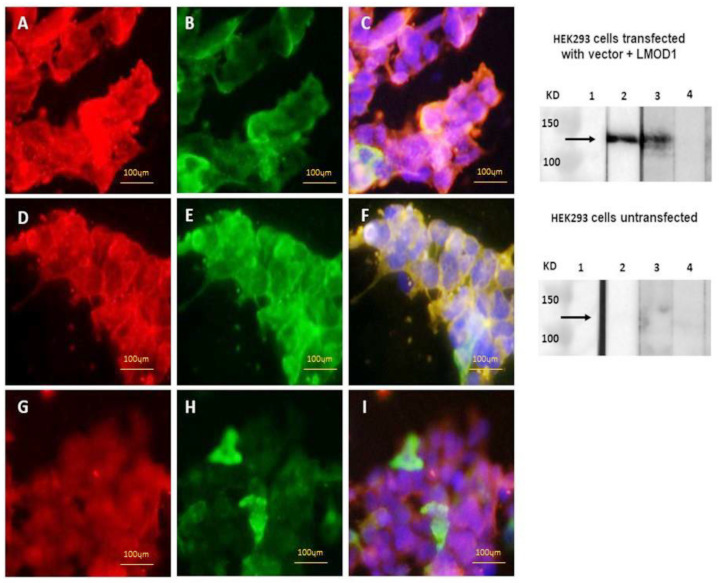
Detection of leiomodin-1 antibodies by cell-based assay (left panel) and Western blot (right panel). (**A**–**C**): cell-based assay with a positive sample; (**D**–**F**): cell-based assay with a negative sample; (**G**–**I**): cell-based assay showing human IgG staining. Red: human leiomodin-1 antibodies; green: leiomodin-1 transfection with GFP tag; blue: 4′,6-diamidino-2-fenylindool. Right panel: Western blot of the commercial leiomodin-1 antibody (lane 2), a positive sample (lane 3) and a negative sample (lane 4) with transfected HEK293 cells (upper right panel) and untransfected cells (lower right panel) as a negative control.

In total, we analyzed 115 sera by a cell-based assay and Western blot. Leiomodin-1 antibodies were detected in the serum of 6/52 (12%) persons with OAE compared to 14/61 (23%) controls without epilepsy (*p* = 0.113) by a cell-based assay (Table 4 and Table 5, Figure 1). Using Western blot, these antibodies were detected in 23/54 (43%) persons screened with OAE (including in 5/22 [23%] with nodding seizures) and 30/61 (49%) controls without epilepsy (Table 4, *p* = 0.479). We did not observe a significant difference in leiomodin-1 antibody prevalence between cases and controls when considering a double positive test (cell-based assay positive and Western blot positive) or only one of the positive tests (Table 4).

The sera of 96 *O. volvulus*-infected persons were tested by a cell-based assay, of which 17 (18%) tested positive for leiomodin-1 antibodies compared to 3/19 (16%) persons without *O. volvulus* infection (*p* = 0.840). Similarly, of 96 *O. volvulus*-infected persons tested by Western blot, 44 (46%) tested positive for leiomodin-1 antibodies compared to 14 (48%) of 29 persons without *O. volvulus* infection (*p* = 0.587) (Table 5). Furthermore, no leiomodin-1 antibodies were found in 12 CSF samples from persons with OAE, nor in 16 CSF samples from persons with acute-onset neurological conditions. Leiomodin was also not found in the serum of 12 Dutch persons with epilepsy or 4 healthy controls.

In multivariable analysis using a cell-based assay, leiomodin-1 antibodies were more likely to be present in males than females (Table 6). However, using Western blot, only increasing age increased the odds of leiomodin-1 antibodies. No association was found between *O. volvulus* infection or epilepsy status and the presence of leiomodin-1 using a cell-based assay or Western blot (Table 6).

### 3.2. Immunohistochemistry to Screen for Auto-Antibodies and of Post-Mortem Brains of People with OAE Who Died

Sera of 170 persons with epilepsy from onchocerciasis-endemic areas and 152 persons without epilepsy were screened for the presence of auto-antibodies by immunohistochemistry on rat brain slides. These 170 cases and 152 controls were included in several epidemiological studies in the Ituri [12], Bas Uélé [13] and Kwilu [16] provinces of the DRC and Maridi County in South Sudan [3].

**Figure 2:**
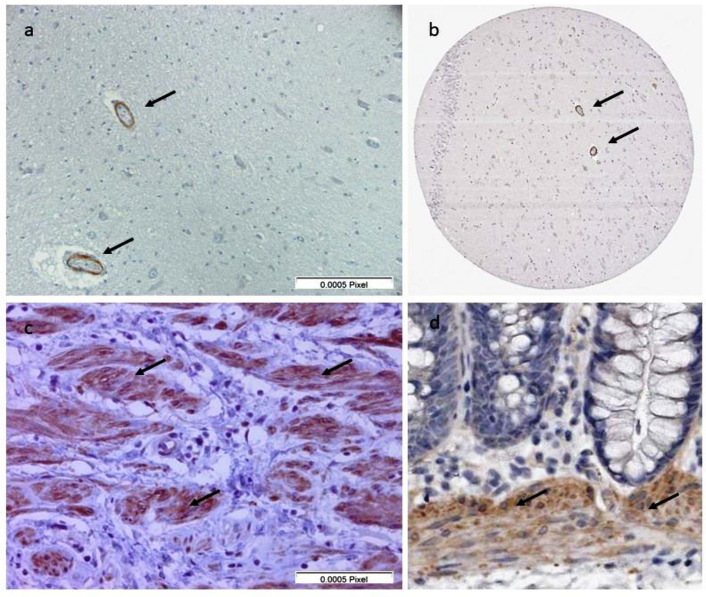
(**a**) Immunohistochemistry of post-mortem brain tissue from the hippocampal area from a person with nodding syndrome. Leiomodin-1 is detected in the blood vessels (black arrows). (**b**) Immunohistochemistry of post-mortem brain tissue from the hippocampal area from the Human Protein Atlas. Leiomodin-1 is detected in the blood vessels (black arrows). (**c**) Immunohistochemistry of colon tissue from this study. Leiomodin-1 is detected in the smooth muscle cells (black arrows). (**d**) Immunohistochemistry of colon tissue from the Human Protein Atlas. Leiomodin-1 is detected in the smooth muscle cells (black arrows).

In none of their sera was any specific staining pattern identified. Immunohistochemistry with commercial leiomodin-1 antibodies on human tissue showed leiomodin-1 staining in the smooth muscle cells of the human colon positive control and in the endothelial cells of the blood vessels in the human post-mortem brain samples (Figure 2). Very weak or non-specific staining was observed in neuronal cells (Figure 2). There was no difference between the brain samples from OAE cases and controls without epilepsy. These results are in accordance with the immunohistochemistry data available in the Human Protein Atlas [9].

## 4. Discussion

Our study does not support the hypothesis that leiomodin-1 antibodies play an etiological role in OAE. Indeed, no significant difference was observed between OAE and controls without epilepsy by the cell-based assay. Moreover, we even detected a lower prevalence of leiomodin-1 antibodies in persons with OAE (10%) compared to controls without epilepsy (23%), although no significant difference was detected in these groups by Western blot, a more sensitive assay in our study. Additionally, leiomodin-1 antibodies were not detected in the CSF of persons with OAE or persons with other neurological conditions. These results are different from an earlier study of Johnson et al., who detected leimodin-1 antibodies in 29/55 (53%) children with nodding syndrome and 17/55 (31%) unaffected village controls (*p* = 0.024), and higher antibody titers in children with nodding syndrome compared to village controls. Moreover, Johnson et al. also detected leiomodin-1 antibodies in the CSF of 8/16 (50%) persons with nodding syndrome compared to none out of 8 North American control persons with epilepsy. Similarly, we also did not detect leiomodin-1 antibodies in any of the Dutch controls in the current study. We cannot exclude that leiomodin-1 antibodies might cross-react with other common parasites co-endemic with *O. volvulus* but absent in American and European populations, such as other filarial parasites or malaria.

Multivariable exact logistic regression did not show an association between *O. volvulus* infection or epilepsy status and the presence of leiomodin-1. This is in contrast with the study findings by Johnson et al. suggesting an association between the presence of leiomodin-1 antibodies and *O. volvulus* infection, and a reaction of leiomodin-1 antibodies with *O. volvulus* lysates [5].

The main differences between the current study and the study by Johnson et al. are the study population and the detection method for leiomodin-1 antibodies. First, Johnson et al. studied Ugandan children with nodding syndrome, whereas we studied Congolese persons with OAE, of which only 27% had a history of nodding seizures. However, these children had similar frequencies of leiomodin-1 antibodies (31%). It has been shown that persons with nodding seizures have a more severe epilepsy and higher microfilarial loads compared to persons with other forms of OAE [20,21]. In addition, Johnson et al. used an enzyme-linked immunosorbent assay (ELISA) to detect leiomodin-1 antibodies, while we used a cell-based assay and Western blot test, the latter being a more specific test for the detection of antibodies than ELISA due to the possibility to control the binding location in the cell and the size of the protein on the blot [22].

Immunohistochemistry using rat brain slices to detect the presence of brain-specific antibodies did not show any specific staining pattern. This method was developed to specifically identify antigenic targets on the membrane of neurons [23]. It has been proven to identify over ten new antibodies mediating autoimmune encephalitis, including those causing seizures [19]. As all samples tested negative, it excludes most of the known antibodies causing autoimmune encephalitis or autoimmune-associated seizures [18]. If positive, it would have guided selection for immunoprecipitation to discover novel antibodies. However, a negative result does not exclude, at all, the presence of antibodies, as human-specific antigens may not be recognized on rat brains, or conformational epitopes may be modified by the fixation techniques. Immunohistochemistry detected leiomodin-1 in the walls of the cerebral blood vessels in post-mortem brain samples of persons with OAE who died, as also presented in the Human Protein Atlas [9,10].

The strength of our study is that we examined a relatively large set of samples of persons with OAE and controls, including both village controls and non-African controls. A weakness is that only a limited number of persons with OAE presented with nodding seizures. In conclusion, leiomodin-1 antibodies do not seem be to play a role in the pathogenesis of OAE. Further research is needed to elucidate the physiopathology of OAE.

## Figures and Tables

**Table 1:** Study participants of whom serum samples were analyzed for presence of leiomodin-1 antibodies.

Region and Type of Study	Study Participants	Number
Ituri Province, Logo health zone, clinical trial [14], case–control study [12,13], REMO study [15]	OAE, skin snip and/or OV16 RDT positive	54
	No epilepsy, *O. volvulus* infection	42
	No epilepsy and no *O. volvulus* infection ^1^	19
Epilepsy centers and academic hospitals, the Netherlands [18]	Focal epilepsy without antibodies associated with encephalitis	12
	Healthy volunteers	4

^1^ Skin snips were not obtained, but men were considered not to be infected with O. volvulus in the absence of subcutaneous Onchocerca nodules together with a negative OV16 RDT.

**Table 2:** Study participants of which cerebrospinal fluid samples were analyzed for the presence of leiomodin-1 antibodies.

Region	Study Participants	Number
Maridi, South Sudan [3]	OAE	12
Mosango hospital, Kwilu Province, DRC [16]	Acute-onset neurological conditions	16

**Table 3:** Selected brain regions for leiomodin-1 immunohistochemistry.

Case 1	Case 2	Case 3	Case 4	Case 5	Control 1	Control 2
Hippocampus (dentate gyrus)	Hippocampus (dentate gyrus)	Hippocampus (dentate gyrus)	Hippocampus (dentate gyrus)	Hippocampus	Hippocampus	Hippocampus
Hippocampus + amygdala	Cerebellum cortex	Hippocampus anterior part 1	Hippocampus	Cerebellum cortex	Cerebellum cortex	Cerebellum cortex
	Cerebellum vermis	Hippocampus anterior part 2	Cerebellum cortex	Cerebellum vermis	Frontal cortex	Frontal cortex
	Frontal cortex	Hippocampus anterior part 3	Cerebellum vermis	Thalamus + corpus mamillaria	Sulcus calcarinus	
	Caudate nucleus	Cerebellum cortex		Hypothalamus anterior + corpus mamillaria		
	Primary motor cortex	Cerebellum vermis		Hypothalamus posterior + corpus mamillaria		
	Parietal cortex			Superior gyrus of the temporal lobe		

**Table 4:** Presence of leiomodin-1 antibodies in serum from persons with OAE and persons without epilepsy from Ituri Province, DRC.

Parameter	Persons with OAE (N = 54) *	Controls without Epilepsy (N = 61)	*p*-Value
Age (median, IQR)	19 (14–23)	31 (15.5–46.5)	<0.001
Male (N, %)	30 (55%)	49 (80%)	0.004
Nodding seizures (N, %)	16	NA	NA
Age of seizure onset (median, IQR)	10 (7–13)	NA	NA
Ever treated with ivermectin (N, %)	4 (7%)	11 (19%)	0.091
Leiomodin-1 antibodies by CBA (N, %)	6/52 (11%)	14 (23%)	0.113
Leiomodin-1 antibodies by WB (N, %)	23/54 (43%)	30 (49%)	0.479
Leiomodin-1 antibodies positive, CBA and WB (N, %)	5/52 (10%)	12 (20%)	0.136
Leiomodin-1 antibodies positive, CBA or WB (N, %)	24/54 (44%)	32 (52%)	0.391

IQR: interquartile range; NA: not applicable; CBA: cell-based assay; * CBA not conducted in two persons; WB: Western blot.

**Table 5:** Prevalence of serum leiomodin-1 antibodies in serum from *O. volvulus*-infected and non-infected individuals.

	*Ov* + (*N* = 96)	*Ov* − (*N* = 19)	*p*-Value
Leiomodin-1 CBA+ (*N*, %)	17 (18%)	3 (16%)	0.840
	*Ov* + (*N* = 96)	*Ov* − (*N* = 19)	
Leiomodin-1 WB + (*N*, %)	44 (46%)	10 (53%)	0.587
Leiomodin-1 antibodies positive, CBA and WB (*N*, %)	17 (18%)	2 (11%)	0.397

*Ov*: *O. volvulus*; CBA: cell-based assay; WB: Western blot.

**Table 6:** Exact logistic regression to assess factors associated with the presence of leiomodin-1 antibody detected by cell-based assay or Western blot.

	Cell-Based Assay				Western Blot			
Parameter	Estimate	95% CI		*p*-Value	Estimate	95%CI		*p*-Value
Gender (Male vs. Female)	3.599	1.271	24.836	0.007	1.352	0.821	2.270	0.278
Age (years)	0.977	0.940	1.014	0.230	1.033	1.003	1.065	0.031
Persons with (OAE vs. control without epilepsy)	0.528	0.237	1.106	0.100	1.142	0.680	1.962	0.757
*O. volvulus*-infected vs. no infection	1.777	0.794	4.672	0.208	1.029	0.525	2.020	0.928

## Data Availability

The datasets generated during the current study are available from the corresponding authors on reasonable request.
